# Investigating the Cellular Distribution and Interactions of HIV-1 Nucleocapsid Protein by Quantitative Fluorescence Microscopy

**DOI:** 10.1371/journal.pone.0116921

**Published:** 2015-02-27

**Authors:** Halina Anton, Nedal Taha, Emmanuel Boutant, Ludovic Richert, Heena Khatter, Bruno Klaholz, Philippe Rondé, Eléonore Réal, Hugues de Rocquigny, Yves Mély

**Affiliations:** 1 Laboratoire de Biophotonique et Pharmacologie, UMR 7213 CNRS, Université de Strasbourg, Faculté de Pharmacie, Illkirch, France; 2 Institut de Génétique et de Biologie Moléculaire et Cellulaire, UMR 7104 CNRS, U964 Inserm, Université de Strasbourg, Illkirch, France; University of South Carolina School of Medicine, UNITED STATES

## Abstract

The nucleocapsid protein (NCp7) of the Human immunodeficiency virus type 1 (HIV-1) is a small basic protein containing two zinc fingers. About 2000 NCp7 molecules coat the genomic RNA in the HIV-1 virion. After infection of a target cell, the viral core enters into the cytoplasm, where NCp7 chaperones the reverse transcription of the genomic RNA into the proviral DNA. As a consequence of their much lower affinity for double-stranded DNA as compared to single-stranded RNAs, NCp7 molecules are thought to be released in the cytoplasm and the nucleus of infected cells in the late steps of reverse transcription. Yet, little is known on the cellular distribution of the released NCp7 molecules and on their possible interactions with cell components. Hence, the aim of this study was to identify potential cellular partners of NCp7 and to monitor its intracellular distribution and dynamics by means of confocal fluorescence microscopy, fluorescence lifetime imaging microscopy, fluorescence recovery after photobleaching, fluorescence correlation and cross-correlation spectroscopy, and raster imaging correlation spectroscopy. HeLa cells transfected with eGFP-labeled NCp7 were used as a model system. We found that NCp7-eGFP localizes mainly in the cytoplasm and the nucleoli, where it binds to cellular RNAs, and notably to ribosomal RNAs which are the most abundant. The binding of NCp7 to ribosomes was further substantiated by the intracellular co-diffusion of NCp7 with the ribosomal protein 26, a component of the large ribosomal subunit. Finally, gradient centrifugation experiments demonstrate a direct association of NCp7 with purified 80S ribosomes. Thus, our data suggest that NCp7 molecules released in newly infected cells may primarily bind to ribosomes, where they may exert a new potential role in HIV-1 infection.

## Introduction

**Fig 1:**
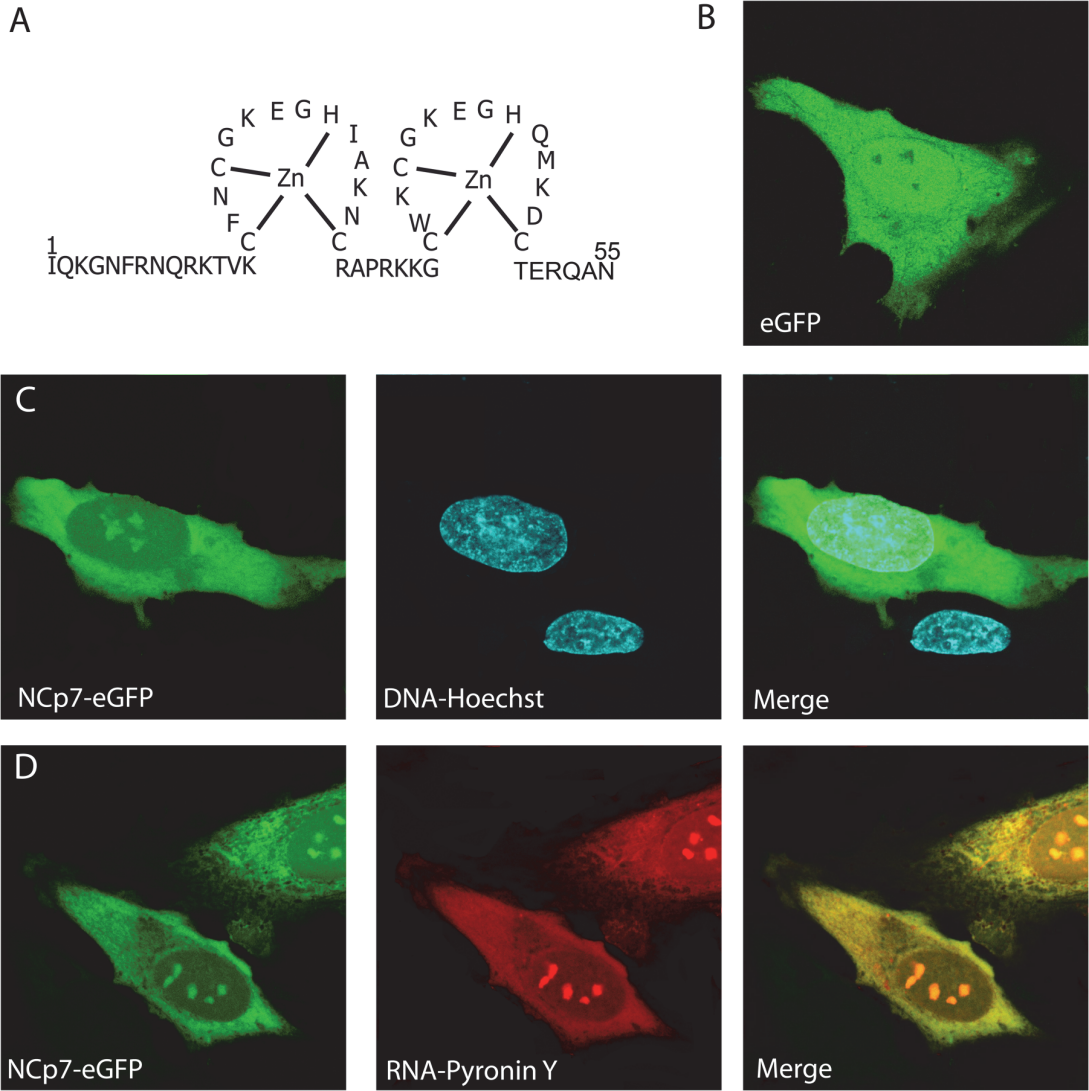
Intracellular distribution of NCp7-eGFP. (A) Amino acid sequence of NCp7. Confocal images of HeLa cells expressing transiently eGFP (B) or NCp7-eGFP (C, D). Comparison with the localization of DNA labeled by 1.6 μM Hoechst 33342, (C) and RNA labeled by 1 μM Pyronin Y. The cyan color of the merge panel in (C) indicates colocalization of NCp7 with DNA in the nucleus. The nearly uniform yellow color of the merge panel in (D) indicates a strong colocalization of NCp7 with RNA all over the cell.

The nucleocapsid protein (NCp7) of the human immunodeficiency virus type 1 (HIV-1) is a small basic protein resulting from the cleavage of the Gag polyprotein precursor by the viral protease. The mature form of NCp7 contains 55 amino acids forming two highly conserved CCHC zinc-finger motifs connected by a short flexible linker and flanked by unfolded N- and C-terminal basic domains (Fig. 1A) [1,2]. In their zinc bound form, the two zinc fingers exhibit similar folding and are in close proximity [2–4].

NCp7 is endowed with key functions in the early and late phases of HIV-1 replication [5–12]. These functions rely on its chaperone properties, which direct the rearrangement of nucleic acids (NAs) into their most thermodynamically stable conformation [13–16]. Within the reverse transcription complex (RTC), NCs are thought to be required for the initiation and the two obligatory strand transfer reactions of viral DNA synthesis by the RT enzyme [8,17–24]. In the nucleus, NCp7 may assist the integrase-mediated integration of the viral DNA into the host cell DNA [25,26]. Later, during virus assembly, the nucleocapsid domain of the Gag polyprotein is responsible for the selection and packaging of the genomic RNA into assembling virions [7,27–29]. Last, in the mature virus the genomic RNA dimer undergoes condensation and is coated and protected by about 2000 molecules of NCp7 in the nucleocapsid substructure [30–33].

Though the mechanism of the chaperoning functions of NCp7 has been widely studied, its trafficking and interactions with cellular components during the early stages of infection are unclear. Once viral DNA synthesis is completed, the RTC becomes the so-called preintegration complex (PIC), which is the integration-competent HIV-1 complex that drives integration of the viral DNA into the host cell DNA. However, the exact composition of the PIC and notably its content in NCp7 as well as the timing of the viral capsid uncoating are debated (for review, see [34]). Based on the much lower affinity of NCp7 for double-stranded DNA than for single-stranded RNAs [17,35,36], it was hypothesized that NCp7 molecules are progressively released during viral DNA synthesis [35,37]. In support of this hypothesis, immunolabeling of NCp7 in H9 cells acutely infected with HIV-1, revealed a diffuse cytoplasmic localization of the protein 4 hours post infection as well as a nuclear localization 18 hours post infection, suggesting that the released NCp7 molecules may diffuse in the cytoplasm and the nucleus [38]. Moreover, NCp7 was also detected in the nucleus 8 hours post infection in infected P4 cells [39]. Due to the large number of NCp7 molecules initially present in the infecting viruses, a significant fraction might be released in the cytoplasm and in the nucleus of the infected cells. It remains that the fate and possible role of these released NCp7 molecules are still unknown.

In this context, in order to characterize the possible intracellular distribution of these released NCp7 molecules and to identify their potential interactants, we used HeLa cells transfected with eGFP-labeled NCp7 as a model. Using a combination of advanced quantitative fluorescence microscopy techniques, we found that NCp7 was distributed in the cytoplasm and the nucleoli where it mainly binds to ribosomal RNAs. Moreover, direct monitoring and mapping of the dynamics of NCp7 and ribosomal proteins further confirmed that NCp7 can bind to ribosomes. Finally, the binding of NCp7 to purified 80S ribosomes was directly demonstrated by gradient centrifugation experiments.

## Materials and Methods

### 1. Constructs

The NC domain of Gag was PCR amplified from the Gag-TC plasmid kindly provided by D. Ott [40]. The PCR product was purified using a PCR purification kit (Macherey Nagel GmbH, Germany), digested with BamH1 and then ligated in pEGFP-N1 to produce NC-eGFP. The integrity of all constructs was confirmed by DNA sequencing (GATC Biotech, Germany).

### 2. Cell Culture and DNA transfections

HeLa cells were cultured on 35 mm glass coverslips (μ-Dish IBIDI, Germany) in Dulbecco's modified Eagle medium supplemented with 10% fetal calf serum (Invitrogen Corporation, France) at 37°C in a 5% CO_2_ atmosphere. Transfection was performed with jetPEI (PolyPlus transfection, France) according to supplier's recommendations. All experiments were done between 16–24 hours post DNA transfection.

The Hoechst 33342, Pyronin Y, Sytox Orange and RNase were purchased at Sigma Aldrich. Staining of nucleic acids was performed by incubating live HeLa cells for 30 minutes with either Hoechst 33342 (1.6 μM) or Pyronin Y (1 μM). For the RICS experiments, the Pyronin Y concentration was 10 nM and the incubation time was reduced to 5 minutes. Due to the high concentrations of RNAs in cells and the rather high binding affinity of Pyronin Y for RNAs [41], only marginal amounts of Pyronin Y molecules are expected to diffuse in their free form in the cells. For Sytox Orange staining, cells were fixed for 15 minutes with 4% PFA solution, then permeabilized using 1% saponin and incubated for 30 minutes with 2.5 μM Sytox Orange. To digest the cellular RNAs, cells were treated for 30 minutes at 37°C by a mixture of RNaseA and RNase T1 (Ambion Rnase cocktail, Life Technologies, France) added at 25 U/mL and 100 U/mL, respectively.

### 3. Linear sucrose density gradient analysis

Human 80S ribosomes were purified from HeLa cells as previously reported [42] and incubated with NCp7 for 30 minutes at 4°C. The mixture was loaded on 15–30% sucrose density gradients prepared in buffer A (20 mM Tris-HCl pH 7.5, 2 mM Mg(OAc)_2_, 150 mM KCl) and centrifuged at 25 000 rpm for 10.5 hours using a SW41 rotor. The gradients were fractionated and fractions corresponding to the peak of 80S ribosomes were precipitated with cold acetone. Proteins in the fractions were separated by SDS-PAGE and probed by western blotting using specific antibodies against NCp7 (polyclonal antibody kindly provided by R. Gorelick), RPS7 (abcam ab57637) and RPL26 (abcam ab59567), and peroxidase-labeled secondary antibody. NCp7, RPS7 and RPL26-specific bands were visualized by luminescence.

### 4. Confocal microscopy and Raster Image Correlation Spectroscopy

Confocal microscopy and Raster Image Correlation Spectroscopy (RICS) experiments were performed on a Leica SP2 microscope equipped with a 63× oil immersion objective (NA = 1.2). eGFP fluorescence was excited with a 488 nm argon laser line and the emitted fluorescence (500–550 nm) was detected by a PMT detector. The RICS analysis was performed using the SimFCS software developed by the Laboratory for Fluorescence Dynamics (http://www.lfd.uci.edu). For each cell, a stack of 50 images (256×256 pixels with a pixel size of 50 nm) was acquired at 400 Hz (2.5 ms between the lines) with a pixel dwell time of 4.8 μs. Moving average subtraction was performed to remove the contribution of slow moving structures and cellular displacements [43]. The average spatial correlation was fitted by a three dimensional diffusion model. A 50 nM solution of eGFP in water was used for calibrating the focal volume, assuming that its diffusion constant D is 90 μm^2^/s [43].

### 5. Fluorescence correlation spectroscopy

FCS measurements were performed on a home-built multiphoton microscope based on an inverted microscope (IX70, Olympus, Japan) [44,45]. The excitation light at 900 nm was provided by a mode-locked Ti:Sapphire laser (Tsunami, Spectra Physics, CA) or a broad band Insight Deep See laser (Spectra Physics, CA) delivering femtosecond pulses at 80 MHz frequency. The laser beam was focused by a 60× (NA = 1.2) water immersion objective (Olympus, Japan). Emitted fluorescence was filtered using a short-pass filter with a cutoff wavelength of 680 nm (F75–680; AHF, Germany) and a band pass filter of 520/17 nm (F37–520; AHF, Germany). The fluorescence was directed to a fiber-coupled APD (SPCM-AQR-14-FC; Perkin Elmer, CA), and the normalized autocorrelation function was calculated on-line with a hardware correlator (ALV5000, ALV GmbH, Germany).

The focal volume was measured prior to experiments using a 50 nM solution of tetramethylrhodamine (TMR) with a known diffusion coefficient (D = 592 μm^2^/s at 37°C) [46]. For each condition, measurements were performed in the cytoplasm and the nucleus of 16 different cells. For each measurement, 50 acquisitions of 5 seconds duration were realized and the autocorrelation curves were calculated. Five averages of ten autocorrelation curves were calculated and analyzed independently by the Quickfit software [47], using the anomalous diffusion model:

$$G(\tau )=\frac{1}{V_{eff}\langle C\rangle }\frac{1}{\left(1+{\left(\frac{\tau }{\tau_{D}}\right)}^{\alpha }\right)}\frac{1}{\sqrt{1+{\left(\frac{r_{0}}{z_{z}}\right)}^{2}{\left(\frac{\tau }{\tau_{D}}\right)}^{\alpha }}}$$ (1)

where *Veff* and <*C*> represent the effective focal volume and the average concentration of fluorescent molecules, respectively while *r*_*0*_ and *zz* correspond to the lateral and axial dimensions of the focal volume, respectively. *τ*_*D*_ corresponds to the transit time and *α* is a coefficient related to the anomalous diffusion. The size of the beam waist in the focal plane was determined by a calibration measurement and the diffusion coefficients were calculated from the transit time values:

$$D=\frac{\omega_{XY}^{2}}{4\tau_{D}}$$ (2)

Noticeably, Equation 2 only rigorously applies in the case of free diffusion [48]. Nevertheless, for the sake of comparison, apparent diffusion constants were calculated with the same equation in case of anomalous diffusion [49].

Fluorescence cross-correlation spectroscopy (FCCS) measurements in live cells were performed on the same setup, according to the recommendations of Bacia et al [50]. An excitation of 1020 nm was provided by a femtosecond laser (Insight, Spectra Physics, CA) source. Fluorescence emission was collected and separated from excitation by a first dichroic (SP 720 nm, Semrock, NY). A second dichroic (LP 585, Chroma, VT) was used to split the emission in green (BP 525–39, Semrock, NY) and red (BP 660–52, Semrock, NY) channels. The filters were selected to minimize the bleed-through of eGFP and mCherry emission between the two channels. The bleed-through was quantified with cells expressing eGFP or mCherry alone. Power intensity was adjusted to optimize the count rate and minimize the noise and saturation effects for cells transfected with eGFP alone, mCherry alone, eGFP-mCherry fusion protein and with eGFP and mCherry, simultaneously. The calibration of the focal volume at 1020 nm was performed prior to experiments using a 50 nM solution of eGFP preliminary characterized at 930 nm by FCS measurements. FCS and FCCS correlation curves were obtained by averaging 60 measurements of 5 s for five different cells. Curves were calculated and analyzed independently by the Quickfit software [47], using the anomalous diffusion model (1). Values of the FCCS parameters were extracted with the Global FCCS Fit plugin of the Quickfit software.

### 6. Fluorescence lifetime imaging microscopy (FLIM)

Time-correlated single-photon counting FLIM was performed on the same two-photon microscope as described for FCS, using an excitation wavelength of 900 nm. Imaging was carried out with a laser scanning system using two fast galvo mirrors (Model 6210, Cambridge technology, MA), operating in the descanned fluorescence collection mode. The fluorescence was directed to a fiber coupled APD (SPCM-AQR-14-FC, Perkin Elmer, CA), which was connected to a time-correlated single photon counting (TCSPC) module (SPC830, Becker & Hickl, Germany), operating in the reversed start-stop mode. Typically, the samples were scanned continuously for about 80 s to achieve appropriate photon statistics to determine the fluorescence decays. Data were analyzed using a software (SPCImage V2.8, Becker & Hickl, Germany), which uses an iterative reconvolution method to recover the lifetimes from the fluorescence decays. The FRET efficiency reflecting the distance between the two chromophores was calculated according to:

$$E=\frac{R_{0}^{6}}{R_{0}^{6}+r^{6}}=1-\frac{\tau_{DA}}{\tau_{D}}$$ (3)

where *R*_*0*_ is the Förster radius, *r* the distance between donor and acceptor, *τ*_*DA*_ is the lifetime of the donor in the presence of the acceptor, and *τ*_*D*_ is the lifetime of the donor in the absence of the acceptor.

### 7. Fluorescence Recovery After Photobleaching (FRAP)

FRAP experiments were performed on an iMIC microscope (Till Photonics, Germany) equipped with a Cobolt Dual Calypso Laser 491/532 nm (Sweden) and a 60× TIRFM (1.45 NA) objective (Olympus, Japan). Fluorescence images were acquired on an EMCCD camera (Andor Tech, Ireland). To avoid possible artifacts of overexpression, only cells expressing low but detectable amounts of protein were chosen for analysis. Briefly, five images were acquired in an epifluorescence configuration before bleach. Photobleaching was done on a selected area in confocal mode by a 488 nm (30% of intensity) laser scanning 30 lines during 30 ms. Recovery was followed in epi configuration at 50 frames per second acquisition rate during 5–10 seconds. For each time point, the intensity of the bleached region was corrected for the bleaching due to the illumination during imaging and the fluorescence intensity was normalized to the prebleached intensity. FRAP recovery curves were generated and fitted using the Offline Analysis (Till Photonics, Germany). The normalized curves were fitted by a double exponential rise model:

$$F(t)=A_{1}(1-e^{-t/\tau_{1}})+A_{2}(1-e^{-t/\tau_{2}})$$ (4)

where A_1_ and A_2_ represent the amplitudes and τ_1_ and τ_2_ the decay times of the two components. The bleached surface S and the values of the recovery halftimes t_1/2_ were used to calculate the diffusion coefficients:

$$D=\frac{S}{4t_{1/2}}$$ (5)

For all measurements, cells were maintained at 37°C.

## Results

### 1. Intracellular localization and binding partners of NCp7-eGFP

Fig. 1 shows typical HeLa cells expressing either eGFP (Fig. 1B) or NCp7-eGFP (Fig. 1C, D). Unlike eGFP which is homogenously distributed all over the intracellular volume, NCp7-eGFP is preferentially localized in the cytoplasm and the nucleoli of the transfected cells. NCp7-eGFP is also observed in the nucleoplasm, but at a smaller intensity than in the other compartments. The distribution differences between NCp7-eGFP and eGFP clearly indicate that the localization of the former is mainly driven by NCp7. A nucleolar localization of NC proteins has been reported for HIV-1 and Rous Sarcoma virus in chicken, mouse and human cells and seems to be a common feature among retroviruses [51]. NC proteins may be retained in the nucleoli through their binding to cellular proteins or RNAs [51]. Moreover, the accumulation of NCp7-eGFP in the nucleoli suggests that it can cross the nuclear envelope, either by diffusion or by an active transport. In the cytoplasm, the distribution is diffuse, indicating that NCp7 does not bind to any specific cytosolic structure or compartment.

Since the biological role of NCp7 is mainly related to its interactions with nucleic acids, we compared its intracellular distribution with that of cellular DNAs and RNAs. Fig. 1C reports cells expressing transiently NCp7-eGFP labeled with Hoechst 33342, a dye that binds specifically to the minor groove of DNA molecules. As expected, we observed a strong signal in the nucleus, due to the high concentration of cellular DNA in this compartment. This pattern does not correspond to the NCp7-eGFP cellular distribution, though from the merge figure, it can be seen that NCp7-eGFP and the Hoechst dye colocalize in the nucleus. Using the RNA specific dye Pyronin Y [52,53], we observed a nearly perfect match of the intracellular distribution of this label with NCp7-eGFP (Fig. 1D) that was confirmed by the uniform yellow color of the cell in the merge panel. This colocalization of NCp7-eGFP with RNA all over the cell suggests that cellular RNAs are the major targets of NCp7-eGFP expressed in HeLa cells. DNA molecules in the nucleoplasm may also be binding partners for NCp7-eGFP, but both the lower accumulation of NCp7-eGFP in the nucleoplasm as compared to the nucleoli and cytoplasm, and the only partial colocalization of NCp7-eGFP with Hoechst suggest that DNA represent a less favored target as compared to RNA. This was expected, since NCp7 exhibits preferential binding to RNAs [17,36], so that binding to cellular RNAs in the nucleoli and cytoplasm is more likely than binding to DNA in the nucleoplasm.

**Fig 2:**
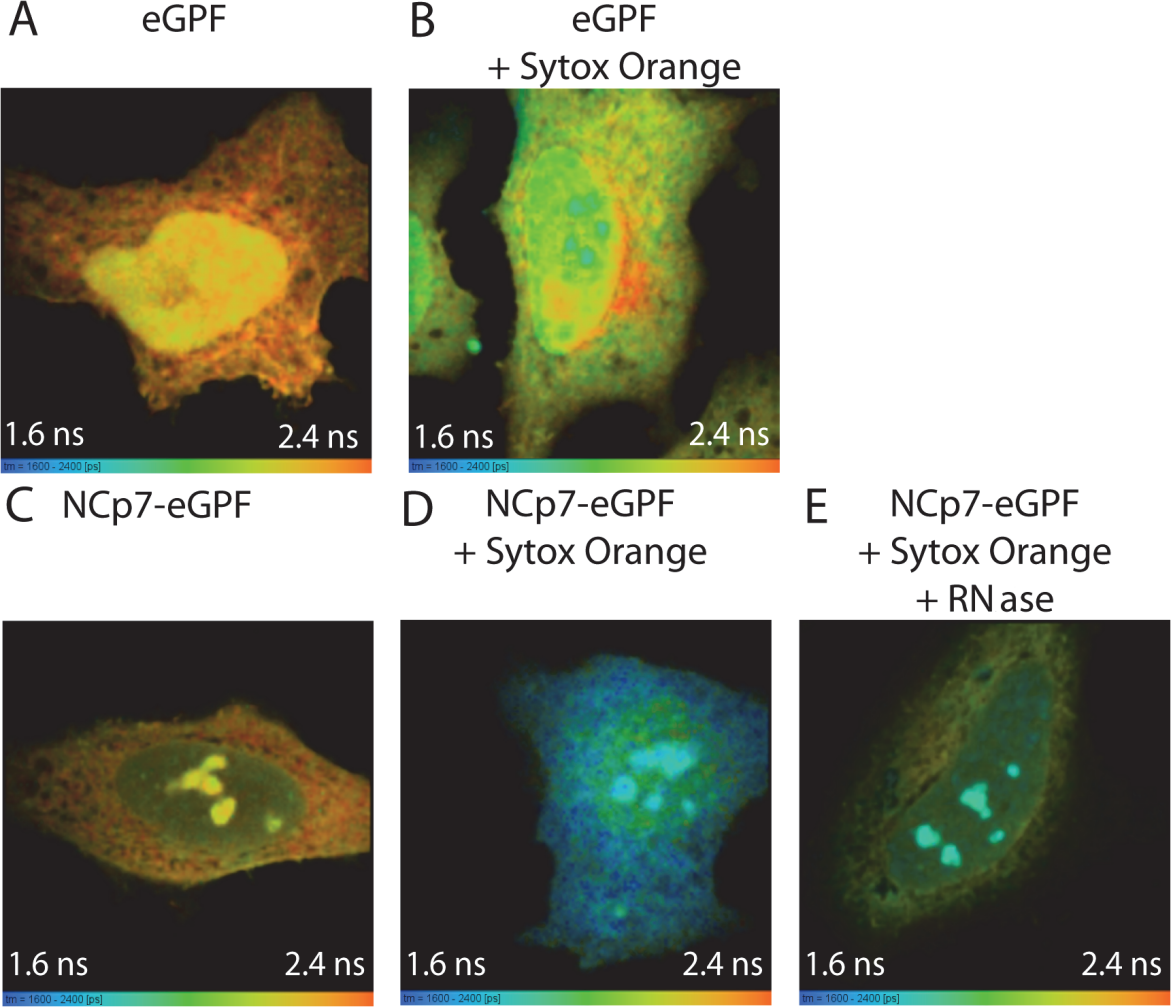
Interaction of NCp7-eGFP with nucleic acids monitored by two-photon FLIM. Fluorescence lifetime color-coded images of eGFP (A, B) and NCp7-eGFP (C, D, E) in the absence (A, C) or in the presence (B, D, E) of 2.5 μM Sytox Orange. In panel E, a mixture of RNaseA and RNase T1 was added at 25 U/mL and 100 U/mL, respectively. The time-resolved decays were fitted with a mono-exponential function. Excitation wavelength was 900 nm. Emission of eGFP was selectively collected through a 515/10 nm filter to remove any contribution from Sytox Orange emission.

In order to further evidence the interactions of NCp7 with cellular RNAs and DNAs, Fluorescence Lifetime Imaging Microscopy (FLIM) was performed to monitor the Förster resonance energy transfer (FRET) between NCp7-eGFP and Sytox Orange, a specific marker of nucleic acids [54]. FRET corresponds to a non radiative energy transfer between a fluorescent donor and an acceptor when they are less than 8 nm apart. This very short distance implies that the labeled molecules interact together, so that FRET can be used to evidence intermolecular interactions [55–57]. In cells, FRET can be followed ideally by the FLIM technique that measures the fluorescent decay at each pixel of the cell image. From these decays, the fluorescence lifetimes (τ) of the FRET donor could be extracted. These lifetimes are absolute parameters that do not depend on the instrumentation or the local concentration of the fluorophores, so they will be only sensitive to FRET. Therefore, the shortening of the fluorescence lifetime of the donor by FRET provides a direct evidence for a physical interaction between NCp7-eGFP and labelled NAs (reviewed in [58]). Sytox Orange was used as a FRET acceptor because its absorption spectrum overlaps the emission of eGFP. However, as this dye does not diffuse across the plasma membrane, FLIM measurements were performed on fixed and permeabilized cells. Noticeably, the permeabilization by saponin caused a slight decrease of the eGFP fluorescence lifetime from 2.43 ns to 2.2–2.3 ns in both eGFP and NCp7-eGFP expressing cells (S1 Table). Staining of eGFP-expressing cells with Sytox Orange caused a less than 5% decrease of the eGFP lifetime in all cell compartments, with the exception of nucleoli, where a 9% decrease was observed (Fig. 2A and 2B). This decrease likely resulted from the high concentration of Sytox Orange molecules bound to the densely packed RNA in the nucleoli, so that the probability to find Sytox Orange molecules close to eGFP molecules gets no more negligible [59]. However, this decrease in the lifetime is small in comparison with the up to 25% decrease in the NCp7-eGFP lifetime observed in the presence of Sytox Orange (Fig. 2C and 2D). Since the autofluorescence of the cells was found to represent less than 1% of the emission of NCp7-eGFP, this large decrease in the lifetime can unambiguously be attributed to FRET, confirming a direct interaction between NCp7-eGFP and labelled NAs. Interestingly, the FRET efficiencies observed in the nucleoli and the cytoplasm (20–25%, Fig. 2D and Table 1) were markedly higher than the FRET efficiency (13%) in the nucleoplasm, confirming that NCp7-eGFP proteins bind less efficiently to DNA as compared to RNA.

**Table 1:** Fluorescence lifetimes and FRET efficiencies for cells expressing eGFP or NCp7-eGFP in the presence of Sytox Orange.

	eGFP	eGFP	NCp7-eGFP	NCp7-eGFP	%	NCp7-eGFP	%
		+ Sytox Orange		+ Sytox Orange	FRET	+ Sytox Orange	FRET
						+ RNAse	
	τ (ns)	τ (ns)	τ (ns)	τ (ns)		τ (ns)	
Whole cell	2.26±0.03	2.16±0.01	2.20±0.06	1.75±0.03	**20**	1.99±0.04	**10**
Cytoplasm	2.30±0.02	2.20 ± 0.02	2.24±0.07	1.69±0.04	**25**	1.97±0.03	**12**
Nucleus	2.20±0.02	2.11± 0.04	2.11±0.05	1.84±0.04	**13**	1.93±0.05	**8.5**
Nucleoli	2.20±0.02	2.0 ± 0.02	2.13±0.07	1.70±0.05	**20**	1.86±0.05	**13**

The lifetime values were extracted from FLIM color coded images such as in Fig. 2, and given as mean +/- SD for 10–13 cells. FRET efficiencies were calculated by comparison with the lifetimes of NCp7-eGFP in the corresponding cell compartments. The SD for the FRET values is about 2%. Cells were fixed with 4% PFA and permeabilized with 1% saponin, as described in Materials and Methods.

To further evidence the NCp7/ RNA interactions, cells were subjected to an RNase treatment to solely remove RNAs (Fig. 2E and Table 1). Results show that the FRET efficiencies dramatically decreased both in the cytoplasm (from 25% to 12%) and the nucleoli (from 20% to 13%). Both the high FRET values and their strong decrease upon RNase treatment confirm that NCp7-eGFP interacts with cellular RNAs in the cytoplasm and the nucleoli. The residual FRET observed after RNase treatment is probably due to complexes of NCp7 bound to incompletely digested RNA fragments or to DNAs. A small decrease in the FRET efficiency was also observed in the nucleoplasm (from 13% to 8.5%). Since DNA should not be affected by the RNase treatment, this FRET decrease may reflect the digestion of nuclear RNAs, such as mRNAs. Noticeably, the presence of incompletely digested RNAs in the nucleus after RNase treatment suggested by the significant signal observed in this compartment (Fig. 2E) could be explained by the rather soft cell permeabilization treatment based on saponin. As previously reported [60], this detergent destabilizes cholesterol containing membranes and is thus not very efficient for nuclear membrane permeabilization. As a consequence, the RNase molecules may not efficiently enter into the nucleus and may thus incompletely digest the nuclear and nucleolar RNAs.

Taken together, the data of Fig. 1 and Fig. 2 indicate that cellular RNAs are likely the major binding partners of NCp7 in the cytoplasm and nucleoli. This conclusion is consistent with the observation that HIV-1 particles contain in addition to the genomic RNA, representing about half of the total RNA, an heterodisperse assortment of cellular RNA species, which are believed to be encapsidated by the NC domain of Gag, in proportion to their cellular level [61].

### 2. Intracellular dynamics of NCp7-eGFP

To further characterize NCp7-eGFP in cells, we performed a series of quantitative fluorescence microscopy experiments in order to explore its intracellular dynamics. In a first step, FRAP measurements were performed to determine the fraction of NCp7-eGFP molecules that are mobile in the intracellular environment and to obtain a first estimation of the NCp7-eGFP diffusion rates in comparison to eGFP alone [62].

**Fig 3:**
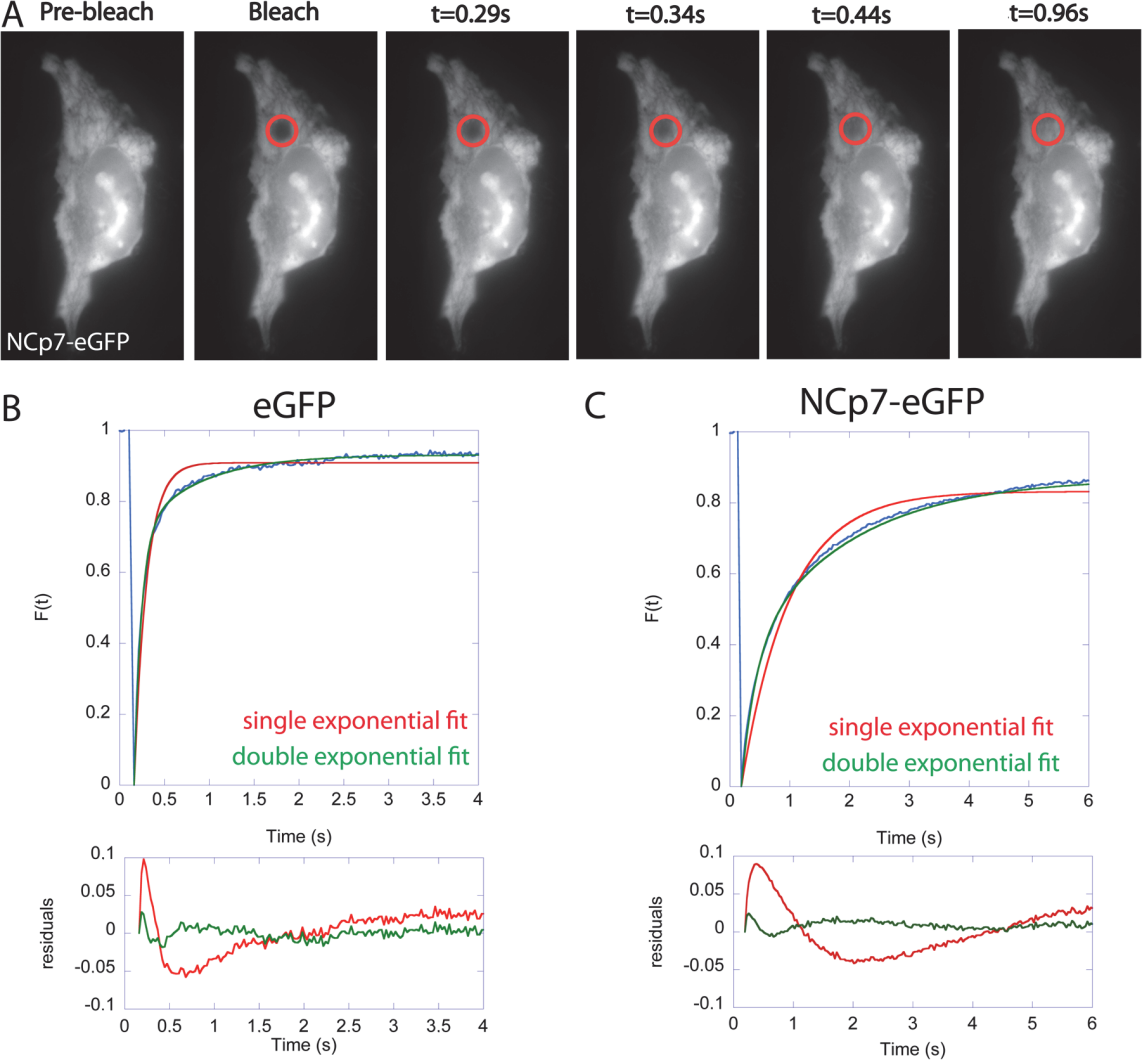
FRAP experiments in eGFP and NCp7-eGFP expressing cells. (A) Time lapse sequence of typical FRAP measurements in the cytoplasm of an NCp7-eGFP expressing cell. The bleached region is highlighted by the red circle. Normalized fluorescence recovery curves of (B) eGFP and (C) NCp7-eGFP in the cytoplasm. For both eGFP and NCp7-eGFP, the distribution of residuals indicated a much better fit of the recovery curves with a double exponential as compared to a single exponential fit.

Approximately 5 μm^2^ areas in the cytoplasm and in the nucleus of eGFP and NCp7-eGFP expressing cells were photobleached by a 488 nm laser (Fig. 3A). A time lapse sequence of 5–10 seconds was acquired just after the bleaching in order to monitor the fluorescence recovery. Representative experimental curves for eGFP and NCp7-eGFP are shown in Fig. 3B and 3C, respectively. As shown by the distribution of the residuals, a double exponential fit (Equation 4 in Materials and Methods section) was required to adequately fit the recovery curve. This bi-phasic shape of FRAP curves in the cellular environment is usually interpreted as a consequence of molecular crowding and presence of obstacles [63–65]. The recovery curves show that the diffusion of eGFP is much faster than that of NCp7-eGFP. The halftime of fluorescence recovery t_1/2_ corresponds to 0.07s and 0.32 s for eGFP and NCp7-eGFP, respectively.

**Fig 4:**
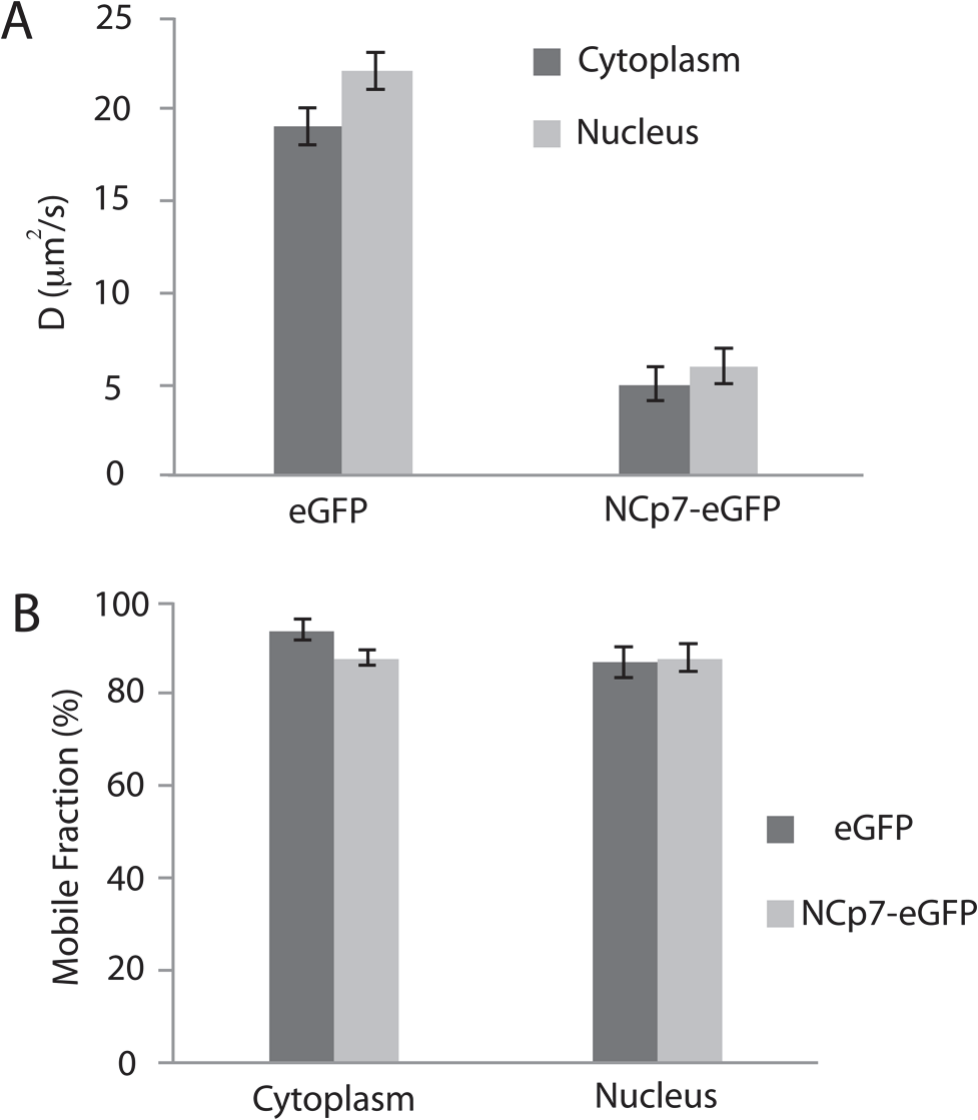
FRAP-based estimation of diffusion coefficient values (A) and mobile fraction (B) of eGFP and NCp7-eGFP in the cytoplasm and in the nucleus.

The values of t_1/2_ were used to estimate the diffusion coefficients according to Equation 5 in Materials and Methods section. It should be noted that it represents only a rough estimation, due to the experimental conditions (bleaching in confocal mode, observation in epi illumination) and the oversimplified 2D diffusion model used for fitting. In spite of these limitations, the intracellular D value (∼20 μm^2^/s, Fig. 4A) determined for eGFP was in good agreement with the values reported in the literature [66,67]. The diffusion rate constant of NCp7-eGFP was found to be ∼4 times slower than that of eGFP. As NCp7 is a relatively small protein (Fig. 1A), its fusion to eGFP causes only a small increase of the hydrodynamic radius from 2.77 nm for eGFP alone [68] to 3.04 nm for NCp7-eGFP. This increase represents less than 10%, so that the 4-fold difference in the diffusion kinetics of these two proteins likely results from the interaction of NCp7 with cellular partners.

Another key information provided by FRAP measurements is the mobile fraction (MF) of NCp7-eGFP molecules that can be deduced from the fluorescence level reached at equilibrium after recovery. MF represents the percentage of fluorescent molecules that diffuse and thus exchange with the photobleached ones. On the opposite, the proportion of NCp7-eGFP molecules bound to static or slowly moving cellular structures can be deduced from the immobile fraction (1-MF). As shown in Fig. 4B, the MF of eGFP (representing freely diffusing control molecule) is about 95% in the cytosol and 87% in the nucleus. Very similar percentages of MF were obtained for NCp7-eGFP in both the cytoplasmic (87%) and nuclear compartments (88%), indicating that NCp7-eGFP molecules form mobile complexes with their cellular partners and notably RNAs, and that only a small fraction (if any) of the NCp7-eGFP molecules are bound to immobile structures.

**Fig 5:**
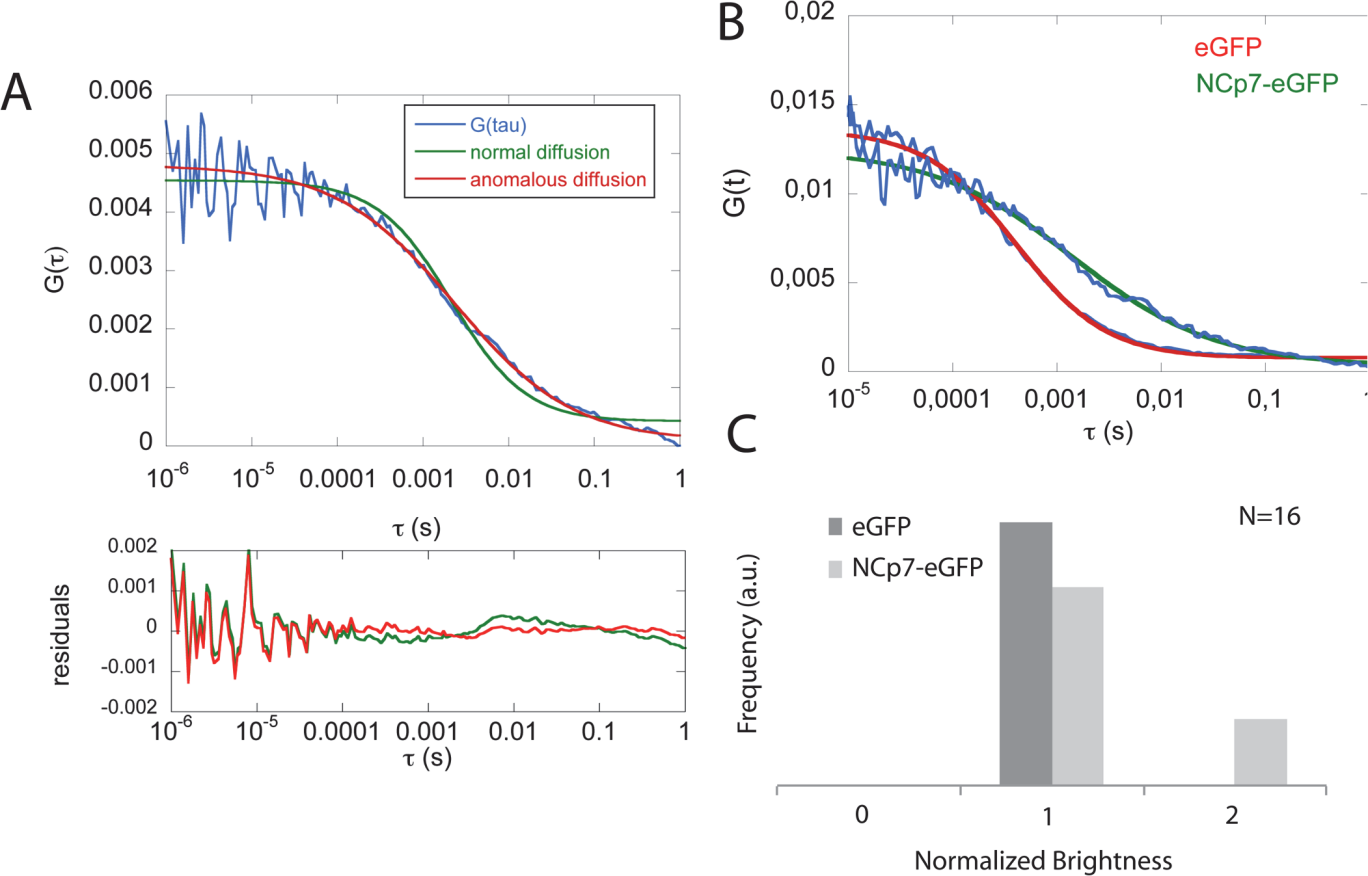
FCS measurements in eGFP and NCp7-eGFP expressing HeLa cells. (A) Experimental autocorrelation function (blue) of NCp7-eGFP in HeLa cells fitted with a model for free (green) and anomalous (red) 3D diffusion. The residuals indicate that a better fit was obtained with the anomalous diffusion model. (B) Comparison of autocorrelation curves for eGFP and NCp7-eGFP diffusion in the cytoplasm of HeLa cells. Fits (solid lines) were performed with the anomalous diffusion model. (C) Histogram of the brightness analysis for eGFP and NCp7-eGFP (N = 16).

To more accurately characterize the intracellular dynamics of NCp7-eGFP, we performed FCS [69–72] and RICS [43,66,73] measurements. Both methods characterize the translational diffusion of fluorescent molecules (or molecular complexes). They are based on the analysis of fluorescence fluctuations during the diffusion of fluorescent molecules through the focal volume of the microscope. FCS provides information about the diffusion time and mode, the local concentration, and about the molecular brightness of the diffusing fluorescent species. The intensity profile of the fluorescence signal at different locations in cells expressing eGFP or NCp7-eGFP was recorded over time and was analyzed by an autocorrelation function (Fig. 5). The autocorrelation curves were adequately fitted with a 3D anomalous diffusion model with one population (Equation 1 in Materials and Methods section). The fit was significantly better than with a free 3D diffusion model with one component, as shown by the residuals (Fig. 5A). Alternatively, a good fit was also obtained with a free 3D diffusion model with two populations, corresponding to free and bound NCp7-eGFP molecules (data not shown). In this case, the fraction of free NCp7-eGFP molecules did not exceed 5%, suggesting that the vast majority of NCp7-eGFP molecules are bound to cellular components, Moreover, the diffusion constant of the slow component was found to be very similar to the diffusion coefficient obtained with the anomalous diffusion model (data not shown).

The autocorrelation curves of NCp7-eGFP were then compared with those of the freely diffusing eGFP protein (Fig. 5B). For eGFP, the ratio of its diffusion coefficient (D) in the intracellular environment to the one in water (D_0_) was found to be 0.306 for the cytosol and 0.280 for the nucleus, in good agreement with previously reported values in HeLa cells, swiss 3T3 fibroblasts and MCKD cells [74–76]. For NCp7-eGFP, its D value in the cytoplasm (4.5 ± 1 μm^2^/s, Table 2) was found to be 8 times slower than the eGFP one (34 ± 3 μm^2^/s). Similarly, a 5.5-fold difference was observed between the corresponding D values in the nucleus. These large differences between NCp7-eGFP and eGFP clearly confirmed that NCp7-eGFP molecules diffuse in the cell in the form of complexes of rather large molecular weight as compared to eGFP molecule. Moreover, the anomalous diffusion coefficient α was found to be close to 1 for eGFP, which indicates that eGFP molecules diffuse in a regime close to free diffusion both in the cytoplasm and the nucleus. For NCp7-eGFP, the average α value is 0.65 ± 0.03 in the cytosol and 0.60 ± 0.09 in the nucleus, indicating that the motion of the NCp7-containing complexes is substantially slowed down either by the obstructed environment [77] or by transient binding events to cytoplasmic or nuclear components [78].

**Table 2:** Diffusion coefficients (D) and anomalous coefficients (α) inferred from FCS and RICS measurements of eGFP and NCp7-eGFP expressing cells.

Cytoplasm			Nucleus		
		D (μm^2^/s)	α	D (μm^2^/s)	α
**FCS**	eGFP	34 ± 3	0.92 ± 0.08	31 ± 1	0.95 ± 0.09
	NCp7-eGFP	4.5 ± 1	0.65 ± 0.03	7 ± 3	0.60 ± 0.09
**RICS**	eGFP	28 ± 3		25.5 ± 2	
	NCp7-eGFP	3 ± 1		5 ± 2	

The D and α values are given as means +/- SD for 800 correlation curves in 16 cells (FCS) and 40 measurements in 10 cells (RICS).

Moreover, FCS curves allowed us to further determine the brightness of these complexes, which is obtained by dividing the average number of photons emitted per second by the average number of fluorescent species diffusing through the focal volume. Comparing this value with the brightness of the eGFP molecules (considered as monomers) measured in the same conditions, helped us to determine the number of NCp7-eGFP molecules in the diffusing complexes. Brightness analysis (Fig. 5C) showed that the complexes do not contain more than two NCp7-eGFP molecules, clearly excluding that the complexes correspond to high molecular weight NCp7-eGFP aggregates.

Taken together, our data suggest that NCp7-eGFP molecules diffuse in both the nuclear and the cytoplasmic compartments in the form of large complexes that contain only one or two NCp7-eGFP molecules.

**Fig 6:**
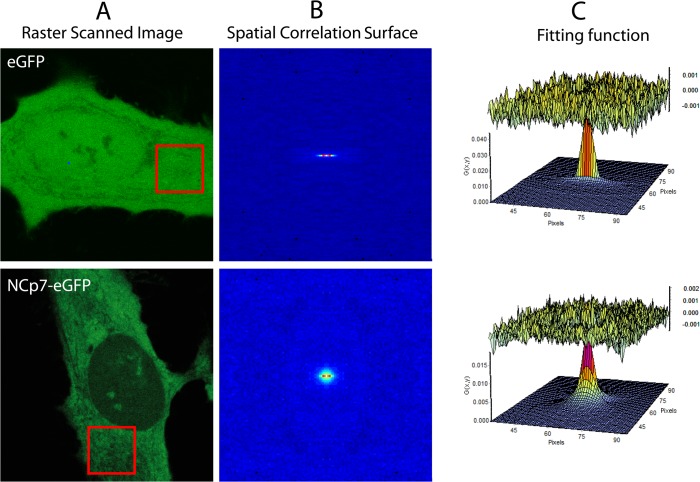
NCp7-eGFP dynamics in HeLa cells monitored by RICS. (A) A series of confocal images of eGFP and NCp7-eGFP expressing cells was acquired. A 128x128 pixel region was analyzed by calculating the two-dimensional spatial autocorrelation function represented as a spatial correlation surface (B) that was fitted by a 3D diffusion model (C), revealing the values of the diffusion coefficients and the number of diffusing molecules.

In order to map the spatial diffusion of NCp7-eGFP, FCS measurements were completed by RICS measurements. RICS analyses the fluorescence intensity fluctuations between neighboring pixels by spatially autocorrelating the image in x and y directions [66]. The fluorescence signal is acquired while the laser beam scans a region of the cell (Fig. 6A), generating a stack of 50–100 images. Afterwards, an average spatial correlation surface of these images is calculated (Fig. 6B) and fitted with a 3D diffusion model (Fig. 6C) in order to obtain information about the diffusion and the concentration of fluorescent molecules. Because the pixel dwell time (∼μs) is much shorter than the time period between two lines (∼ms), the amplitude of the spatial correlation surface (SCS) in the x direction reflects fast diffusion processes. Corresponding amplitudes appear in the y direction, when slowly diffusing molecules are present. As expected, the SCS of the rapidly diffusing eGFP molecules has significant amplitude only in the x direction, while for NCp7-eGFP, it broadens in the y direction, confirming the slower motion of the NCp7-containing complexes (Fig. 6B). The main advantage of RICS compared to FCS for cellular measurements is its lower sensitivity to photobleaching. Since the laser beam is scanning during the acquisition, the illumination time of the excited molecules is much shorter compared to FCS (μs versus ms). The average values of diffusion coefficients measured for eGFP and for NCp7-eGFP by RICS are summarized in Table 2 and are in full agreement with the FCS data, confirming that NCp7-eGFP molecules bind to its cellular partners within large complexes.

**Fig 7:**
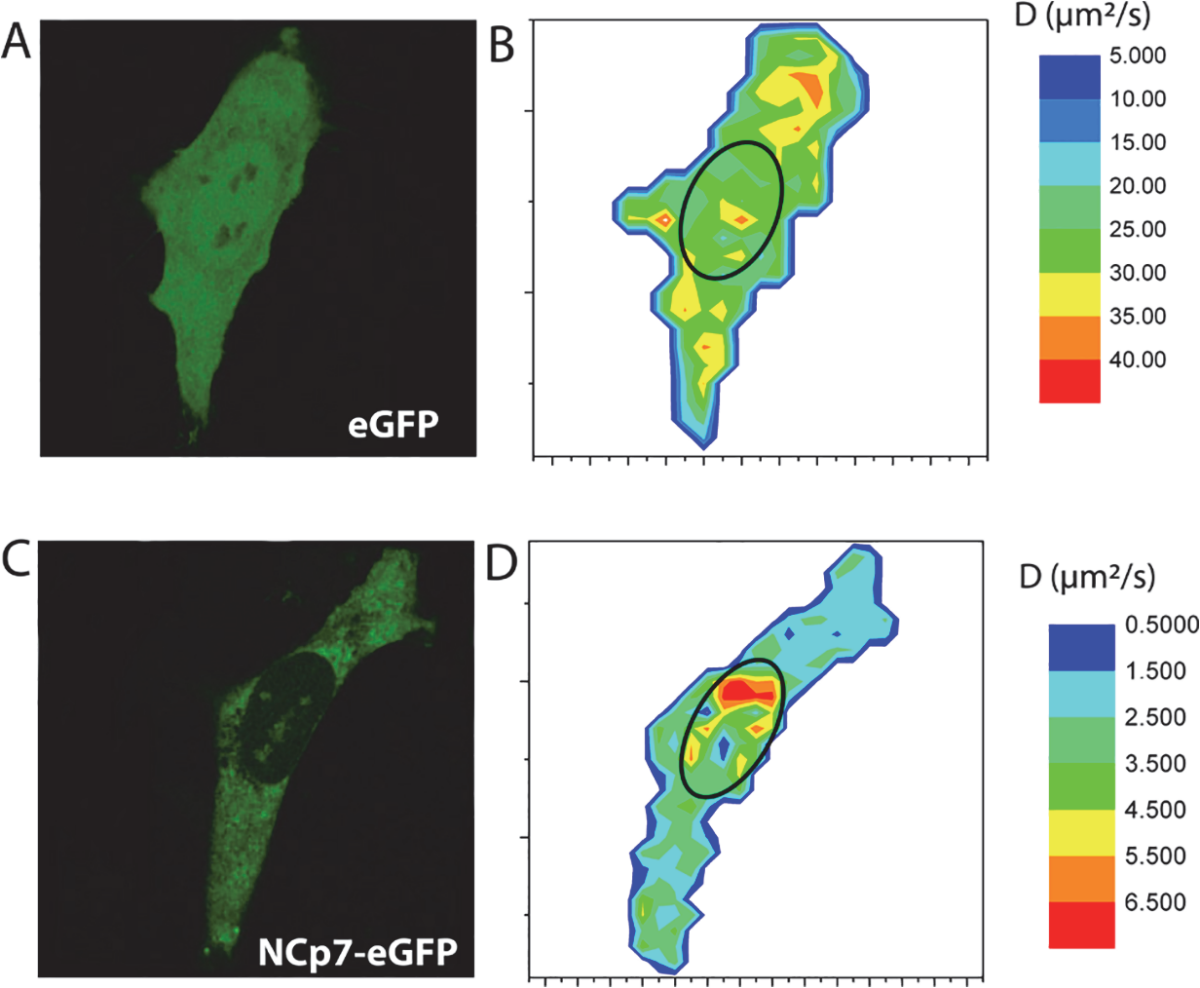
Confocal images (A, C) and RICS-based diffusion maps (B, D) of eGFP and NCp7-eGFP in HeLa cells. The color coded images (B, D) represent the values of the diffusion coefficients measured in the cell. The blue colors at the cell borders are artifacts, due to averaging with the exterior of the cells. Confocal images for the same cells (A, C) are given to identify the cell compartments and contour.

Another advantage of the RICS approach is the possibility to choose the size of the analyzed area. The lower size limit of this area is given by the distance travelled by the protein during the scan and corresponds to 2 μm^2^ (40x40 pixels) for the fast moving eGFP. Consequently, in a stack of images of 1024x1024 pixels, we calculated the D values in windows of 64x64 pixels to draw the diffusion maps of eGFP and NCp7-eGFP in HeLa cells (Fig. 7). For eGFP, the diffusion coefficients are in the range of 20–40 μm^2^/s, with a large majority of values being comprised between 25 and 35 μm^2^/s, in line with previous reports [67]. The distribution of D values was rather homogeneous all over the cell, with no obvious deviation for any compartment. For NCp7-eGFP, the overall D values were much lower and differences in the D values between cell compartments appeared. In the cytoplasm, the diffusion D values were homogeneously distributed between 1.5 and 4 μm^2^/s all over the cytoplasm, thus no specific region of lower or higher mobility could be evidenced. The diffusion in the nucleus was found to be faster and more heterogeneous than in the cytoplasm, being comprised between 3.5 and 6.5 μm^2^/s. Preliminary tracking experiments suggested that these rather high D values in the nucleoplasm may be related to a directed transport of the NCp7-containg complexes in this compartment (data not shown). Further studies are in due course to clarify the mechanism of this directed motion. In contrast, the D value drops to ∼0.5 μm^2^/s in the nucleoli, indicating that the NCp7-eGFP molecules probably form larger complexes with cell partners in the nucleoli than in the nucleoplasm or that the NCp7-containing complexes are further slowed down by the dense and compact environment of the nucleoli.

As NCp7 molecules were found to be largely associated with RNAs in the cytoplasm (Figs. 1 and 2), we next compared the cytoplasmic diffusion of NCp7-eGFP with the diffusion of cellular RNAs labeled with Pyronin Y. The average diffusion coefficient of cytoplasmic RNAs (5.8 +/- 1 μm^2^/s) is significantly higher than the NCp7-eGFP one (3.2 +/- 0.9 μm^2^/s), suggesting that NCp7-eGFP does not bind randomly to all cellular RNAs but interacts preferentially with large RNA molecules and/or with RNA molecules within ribonucleoprotein complexes. Since ribosomal RNAs represent about 80% of the total RNA in HeLa cells [79], it was tempting to speculate that NCp7 binds rRNAs in ribosomes. To further test this hypothesis, we measured the diffusion constant of the ribosomal protein L26 (RpL26), taken as an example. L26 is located at the surface of the large ribosomal 60S subunit. Interestingly, the D values (4.0 +/- 1.5 μm^2^/s) of RpL26-eGFP were close to those of NCp7-eGFP, strengthening the hypothesis that NCp7-eGFP binds RNAs in ribosomes.

**Fig 8:**
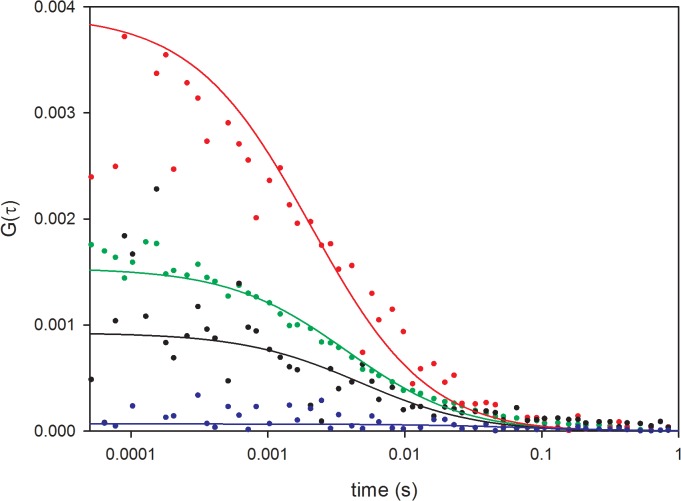
FCCS measurements on HeLa cells expressing RpL26-eGFP and NCp7-mCherry. The green, red and black curves denote the autocorrelation curve of RpL26-eGFP in the green channel, the autocorrelation curve of NCp7-mCherry in the red channel, and the cross-correlation curve between the two channels, respectively. As a negative control, the blue curve corresponds to the cross-correlation between eGFP and mCherry proteins co-expressed in HeLa cells. The close to zero value of the FCCS curve of the negative control not only shows that the two fluorescent proteins do not diffuse together, but also that there is marginal spectral bleed-through between the green and red channels. The solid lines correspond to the fit of the curves to the anomalous 3 D diffusion model. Diffusion constants of 5.6 (+/- 0.7) μm^2^/s, 6 (+/- 3) μm^2^/s and 4 (+/- 3) μm^2^/s were obtained for RpL26-eGFP (green), NCp7-mCherry (red) and the RPL26-eGFP/NCp7-mCherry complex (black), respectively.

To further confirm the interaction between NCp7 and ribosomes, FCCS experiences were performed on HeLa cells co-transfected with RpL26-eGFP and NCp7-mCherry. The FCCS technique correlates the temporal fluorescence fluctuations coming from two differently labeled molecules diffusing through the small sample volume defined in our case by a two-photon excitation. In the absence of spectral bleed-through, the cross-correlation function is different from zero only if both labeled proteins diffuse together, which implies that they belong to the same complex. A typical FCCS curve recorded on cells co-expressing RpL26-eGFP and NCp7-mCherry is shown in Fig. 8 (black trace), showing that the two proteins diffuse together and thus, belong to the same complex. It should be noted that these results show only that the two proteins are parts of the same ribonucleoprotein complex but not that they directly interact together. Quantitative analysis of FCCS curves revealed that the complexes containing both labeled proteins diffuse with a diffusion constant of 4 (+/- 3) μm^2^/s, fully consistent with the diffusion constants of the NCp7-eGFP and RpL26-eGFP proteins measured by RICS. Furthermore, comparative analysis of the FCCS curve (Fig. 8, black curve) with the autocorrelation curves of NCp7-mCherry (Fig. 8, red curve) and RpL26-eGFP (Fig. 8, green curve) shows that between 40 to 70% of NCp7-mCherry molecules cross-correlate with RpL26-eGFP. Thus, the FCCS data clearly indicate that NCp7 and RpL26 diffuse together in the cytoplasm within the same ribosomal complex.

**Fig 9:**
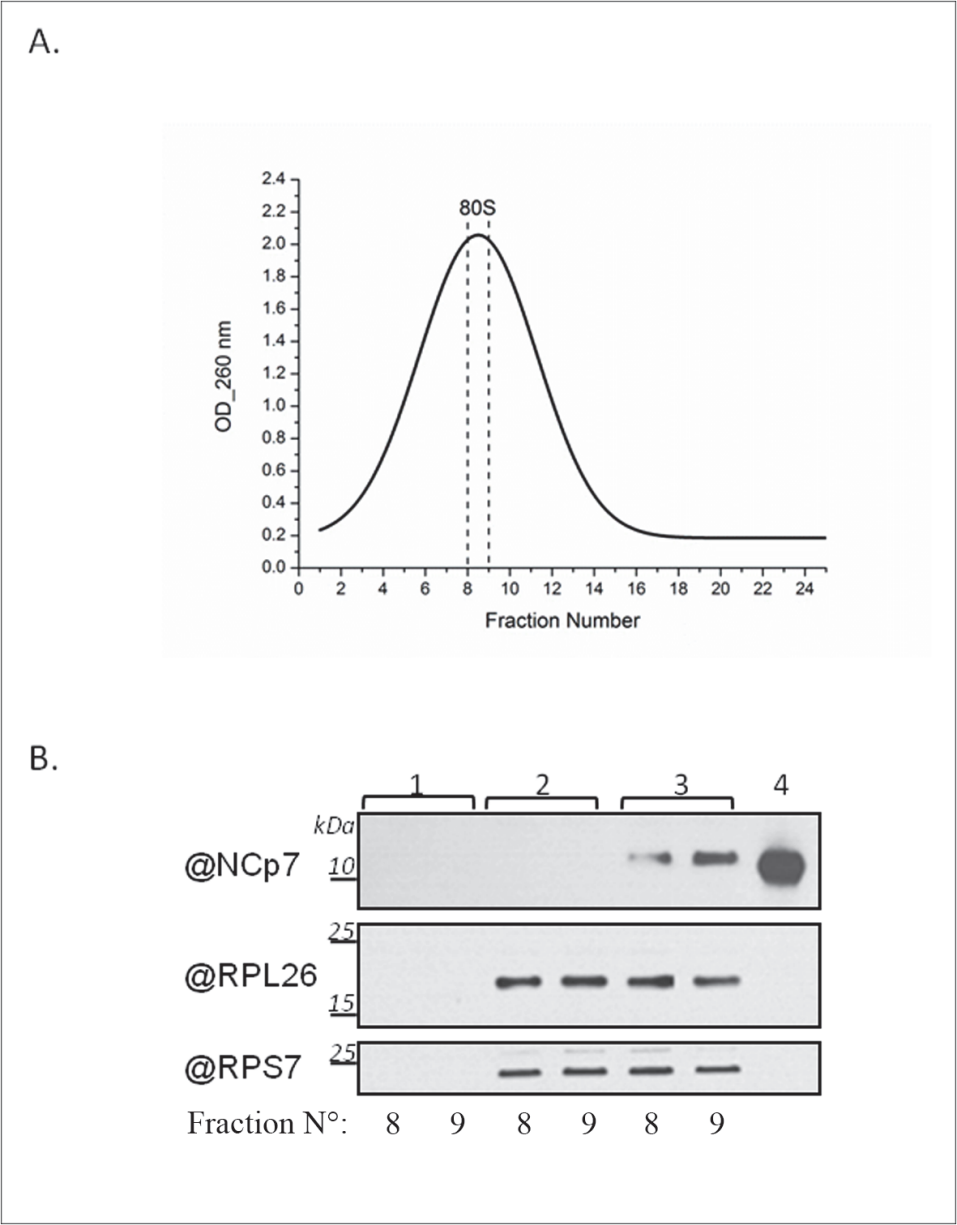
NCp7 cosediments with 80S ribosomes. (A) Sucrose gradient fractionation profile of purified 80S ribosomes (0.9 μM) incubated with NCp7 (13.3μM). Ribosome/NCp7 ratio was about 1/15. The peak fractions (8–9) were precipitated and further analyzed by western blot. (B) Western blot of fractions 8–9 from sucrose gradient fractionations performed with only NCp7 peptide (lanes 1); only 80S ribosomes (lanes 2); and 80S ribosomes and NCp7, together (lanes 3). NCp7 and ribosomal proteins were detected with polyclonal NCp7 and RpS7 antibodies, and monoclonal RpL26 antibodies. As a control, 90 nM of NCp7 peptide was loaded (4).

Finally, we performed gradient centrifugation experiments to directly assess whether NCp7 associates with purified 80S ribosome (Fig. 9). Purified human ribosomes were incubated in the absence or in the presence of purified NCp7 and submitted to fractionation experiments on a linear 15–30% sucrose gradient. The central peak fractions (Fig. 9A) were collected and analyzed by western blot. Antibodies directed against RpS7 and RpL26 proteins, present in the small and large ribosomal subunit, respectively were used to detect the ribosomes, while NCp7 was detected with a polyclonal antibody. A strong cosedimentation of NCp7 and ribosomal proteins (Fig. 9B, compare lanes 3 to lanes 1 and 2) was observed when NCp7 was incubated with the ribosomes prior to centrifugation, demonstrating a clear association of NCp7 with the 80S ribosome.

## Discussion

NCp7 is a nucleic acid chaperone protein with key functions in the early and late phases of HIV-1 replication. Its implications in the reverse transcription process are extensively documented (for reviews, see [12,17,22,80]), but only little is known on NC's fate following the completion of viral DNA synthesis. A model was recently proposed in which due to its lower affinity for dsDNA as compared to ssDNA and vRNA, a large fraction of NCp7 is believed to be released in the cytoplasm during viral DNA synthesis by RT, so that only a small fraction of NCp7 molecules remains associated with the PIC to activate viral DNA integration by the IN enzyme [35,37]. This model is supported by the fact that NCp7 is present at a low level in the nucleus of newly infected cells and barely detectable in the PIC by biochemical techniques [25,38,39,81]. The intracellular distribution and biological role of NCp7 molecules released from the incoming viral particles in newly infected cells are yet totally unknown. In an attempt to explore the fate and possible roles of NCp7 dissociating from the incoming virions, we used a model system based on the expression of eGFP-labeled NCp7 in HeLa cells to study by a combination of fluorescence microscopy techniques the intracellular distribution and dynamics of labeled NCp7, in order to identify its possible intracellular ligands.

Confocal microscopy (Fig. 1) showed that eGFP-NCp7 mainly localizes in the cytoplasm and the nucleoli. The presence of NCp7 in the nucleoli and to a lower extent in the nucleoplasm, is in line with the nuclear localization of NCp7 after infection [38,39] and the possible chaperoning role of NCp7 in vDNA integration by the viral integrase [32,82–84]. In agreement with the data reported by Lochmann et al. [51], our results show also that NCp7 can enter into the nucleus independently of the PIC. Colocalization studies further revealed a nearly perfect match of the intracellular distribution of NCp7 and cellular RNAs. In addition, the high FRET efficiency observed by FLIM between Sytox Orange labeled nucleic acids and NCp7-eGFP together with the large drop of this FRET efficiency upon RNase treatment (Fig. 2) clearly indicate that NCp7 directly interacts with cellular RNAs both in the cytoplasm and the nucleoli. Thus, cellular RNAs appear to be the major intracellular ligand of NCp7. This high tropism for cellular RNAs may well explain why about half of the total virion RNA consists of cellular RNA species, probably packaged via the NC domain of Gag [61]. The binding of NCp7 to cellular RNAs is likely poorly specific since analysis of the composition of the cell RNA species in the virions revealed that they were roughly in proportion to their level in the cell [61].

The significantly lower amount of NCp7 and lower FRET efficiency in the nucleoplasm, together with the lower sensitivity of the FRET efficiency upon RNase treatment further suggest that NCp7 can also bind to double-stranded DNAs in the nucleus, but less efficiently than to RNAs. This conclusion is in line with the significantly lower affinity for double-stranded DNAs in comparison with single stranded RNA and DNA sequences [17,36]. FRAP (Fig. 4) and FCS (Fig. 5, Table 2) experiments further showed that the intracellular concentration of free NCp7-eGFP was negligible and that nearly all NCp7-eGFP molecules diffused in the form of complexes of rather high molecular weight. Moreover, most of these complexes were found to be mobile, being able to diffuse, while only a limited fraction of these complexes were associated with slowly moving or immobile structures. Interestingly, only one or two molecules of NCp7 per complex were found. This clearly excludes any cooperative binding of NCp7 to its cellular partners and strengthens the hypothesis that NCp7 may bind rather uniformly to RNA molecules in the cell. This low amount of proteins in the complexes may be explained by the high concentration of RNAs in the cell and the fact that in all experiments, we used cells displaying low to moderate expression of NCp7-eGFP. Moreover, the absence of cooperative binding of NCp7 to RNAs is in agreement with *in vitro* measurements showing, at best, a moderate cooperative binding of NCp7 to model RNAs [85,86]. Finally, RICS (Figs. 6–7) and FCCS experiments (Fig. 8) together with sucrose gradient fractionation data (Fig. 9) indicated that NCp7 was associated, in the cytoplasm, with ribosomes, probably via a binding with rRNAs, as the latter represent about 80% of all RNA in HeLa cells [79]. Therefore, as a non specific RNA binding protein, NCp7 will have a strong probability to bind to rRNAs. In addition, a number of ribosomal proteins including RpL26 were identified as NCp7 potential cellular partners in screens based on affinity tagging purification combined with mass spectrometry [87], clearly indicating that they potentially constitute with rRNA, additional binding sites for NCp7 in the ribosomes. Thus, ribosomes appear to be a major target for NCp7. Along this line, the mobile and immobile fractions of complexes seen by FRAP in the cytoplasm may tentatively be attributed to free and membrane-bound ribosomes, while the high concentration of NCp7 in the nucleoli may be related to the high molecular weight precursors of ribosomes in this compartment. Moreover, the binding of NCp7 to these high molecular weight precursors in the crowded environment of the nucleoli may well explain the low diffusion coefficients observed in this compartment. Since in the ribosome biogenesis, the ribosome precursors need to shuttle through the nucleoplasm to reach the cytoplasm, the complexes of NCp7 with these ribosome precursors may belong to the RNase-sensitive population of complexes seen by FRET/FLIM in the nucleoplasm.

In conclusion, our data indicate that NCp7 expressed in HeLa cells shows a binding tropism for RNAs, notably for rRNAs in ribosomes. It can thus be proposed that NCp7 leaving the RTC during and/or after viral DNA synthesis by RT in newly infected cells will first bind to ribosomes. As a consequence, NCp7 may influence protein synthesis, thus contributing to the infection process. Experiments are in progress to further characterize the interactions of NCp7 with ribosomes and ribosomal proteins, in order to tackle the new potential role of NCp7 in HIV-1 infection and replication.

## Supporting Information

S1 Table Effect of fixation and cell permeabilization on eGFP and NCp7-eGFP lifetimes. In the absence of Sytox Orange labeling, the distribution and lifetime values of eGFP are homogeneous all over the nucleus, so that nucleoli cannot be identified. Therefore, only one value is represented for the whole nuclear compartment.
(DOC)
