# Supplementary material for: Investigating the Cellular Distribution and Interactions of HIV-1 Nucleocapsid Protein by Quantitative Fluorescence Microscopy
Source: PLoS One. 2015 Feb 27;10(2):e0116921. doi: 10.1371/journal.pone.0116921 (PMC4344342; doi:10.1371/journal.pone.0116921)
Supplement: S1 Table — In the absence of Sytox Orange labeling, the distribution and lifetime values of eGFP are homogeneous all over the nucleus, so that nucleoli cannot be identified. Therefore, only one value is represented for the whole nuclear compartment. (DOC) [file pone.0116921.s001.doc]

Table S1: Effect of fixation and cell permeabilization on eGFP and NCp7-eGFP lifetimes. In the absence of Sytox Orange labeling, the distribution and lifetime values of eGFP are homogeneous all over the nucleus, so that nucleoli cannot be identified. Therefore, only one value is represented for the whole nuclear compartment.

|  | **eGFP** | **NCp7-eGFP** | **eGFP** | | **NCp7-eGFP** |
| --- | --- | --- | --- | --- | --- |
| **Cell treatment** | 4% PFA | | 4% PFA + 1% saponin | | |
|  | τ (ns) | τ (ns) | τ (ns) | τ (ns) | |
| **Whole cell** | 2.43±0.02 | 2.43±0.01 | 2.26±0.03 | 2.20±0.06 | |
| **Cytoplasm** | 2.43±0.02 | 2.42±0.01 | 2.30±0.02 | 2.24±0.07 | |
| **Nucleus** | 2.42±0.03 | 2.43±0.01 | 2.20±0.02 | 2.11±0.05 | |
| **Nucleoli** |  | 2.40±0.02 |  | 2.13±0.07 | |
